# Multi-Omics Analysis of the Epigenetic Effects of Inflammation in Murine Type II Pneumocytes

**DOI:** 10.3390/ijms26104692

**Published:** 2025-05-14

**Authors:** Jenna A. Fernandez, Qiyuan Han, Andrew T. Rajczewski, Thomas Kono, Nicholas A. Weirath, Alexander S. Lee, Abdur Rahim, Natalia Y. Tretyakova

**Affiliations:** 1 Department of Medicinal Chemistry, University of Minnesota, Minneapolis, MN 55455, USA; fernandez.jenna@mayo.edu (J.A.F.); weira008@umn.edu (N.A.W.); rahim032@umn.edu (A.R.); 2 Department of Biochemistry, Biophysics, and Molecular Biology, University of Minnesota, Minneapolis, MN 55455, USA; hanxx963@alumni.umn.edu (Q.H.); rajcz001@umn.edu (A.T.R.); 3 Research Informatics Services, University of Minnesota, Minneapolis, MN 55455, USA; 4 Department of Chemistry, University of Minnesota, Minneapolis, MN 55455, USA; alexanderlee2025@u.northwestern.edu; 5 Department of Medicinal Chemistry, College of Pharmacy, and the Masonic Cancer Center, University of Minnesota, Minneapolis, MN 55455, USA

**Keywords:** lung, type II cells, inflammation, multi-omics, lipopolysaccharide, mouse model, DNA methylation, DNA hydroxymethylation, gene expression, proteomics

## Abstract

Chronic inflammation plays a central role in the pathogenesis of lung diseases including asthma, long COVID, chronic obstructive pulmonary disease (COPD), and lung cancer. Lipopolysaccharide (LPS) is a potent inflammatory agent produced by Gram-negative bacteria and also found in cigarette smoke. Our earlier study revealed that the intranasal exposure of A/J mice to LPS for 7 days altered gene expression levels in alveolar Type II epithelial cells (AECIIs), which serve as precursors to lung adenocarcinoma and are also preferentially targeted by SARS-CoV-2. In the present work, we employed a comprehensive multi-omics approach to characterize changes in DNA methylation/hydroxymethylation, gene expression, and global protein abundances in the AECIIs of A/J mice following the sub-chronic exposure to LPS and after a 4-week recovery period. Exposure to LPS led to hypermethylation at regulatory elements within the genome such as enhancer regions and expression changes in genes known to play a role in lung cancer tumorigenesis. Changes in protein abundance were consistent with an inflammatory phenotype and also included tumor suppressor proteins. Integration of the multi-omics data resulted in a model where LPS-driven inflammation in AECIIs triggers epigenetic changes that, along with genetic mutations, may contribute to lung cancer development.

## 1. Introduction

Inflammation contributes to the etiology of many human diseases including rheumatoid arthritis [1], Alzheimer’s disease [2], long COVID [3], and cancer [4]. Chronic inflammation of the lungs is of particular concern due to the direct exposure of the lungs to external contaminants [5] as well as the known link between lung inflammation and lung cancer [6]. Pulmonary inflammation is an important factor in COVID-19 morbidity and mortality [7], and anti-inflammatory agents are commonly used in its treatment [8]. Microbial elements such as bacteria, fungi, and microbial toxins contribute to lung inflammation [9]. Lipopolysaccharide (LPS) is a highly abundant structural component of the outer membrane of Gram-negative bacteria, which is released following the destruction of the bacterial cell or during normal outer membrane vesicle trafficking [10]. LPS is a potent endotoxin that serves as a powerful activator of the host immune system, stimulating leukocyte inflammatory cytokine production [11]. Environments with extensive Gram-negative bacterial metabolism are generally high in LPS, and it can induce septic shock and inflammatory responses [12]. Due to its potent inflammatory properties, LPS has been used to model chronic obstructive pulmonary disease (COPD) in mouse models [13,14]. LPS treatment has been shown to increase the size and the multiplicity of lung tumors induced by tobacco-specific nitrosamine NNK [15] and to cause early gene expression changes in the lung [16], identifying inflammation as a significant factor in lung cancer development [17].

Epigenetic deregulation provides a possible link between inflammation and lung cancer. Inflammation is known to deregulate DNA and histone marks [15,16,18,19,20]. Epigenetic changes such as the hypermethylation of tumor suppressor genes are recognized as a hallmark of tumorigenesis [21]. However, to our knowledge, no multi-omics studies have been conducted to characterize the inflammation-induced epigenetic changes in the target cells for smoking-induced adenocarcinoma (Type II cells). With rare exceptions [16], previous studies of inflammation-induced epigenetic changes in the lung have been limited to whole lung tissue and did not investigate inflammation-mediated changes in specific cell types [15,19]. Since inflammatory response triggers the recruitment of macrophages and lymphocytes to the lungs and thus changes the cellular composition of the lung tissue, whole tissue analyses are likely to mask key epigenetic events in target cells [22].

**Figure 1:**
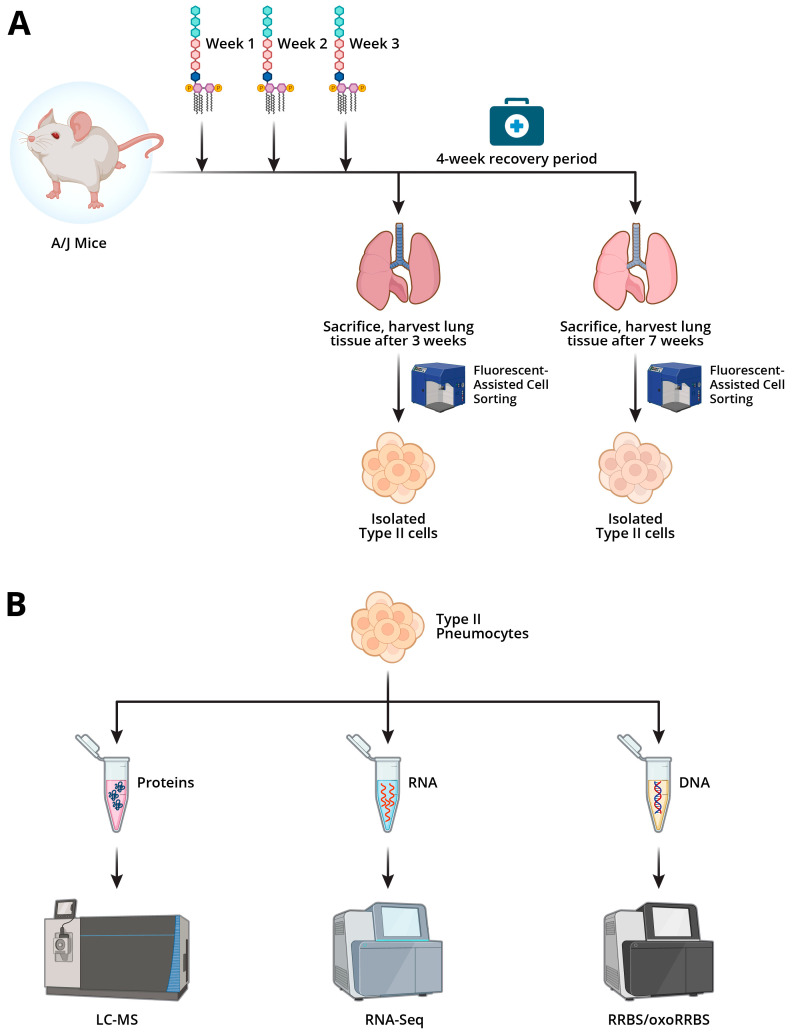
Outline of experimental procedures for multi-omics analyses of LPS induced changes in murine AECIIs: (**A**) LPS exposure scheme. A/J mice were treated intranasally once a week with increasing amounts of LPS or PBS vehicle only. Lungs were harvested, and AECIIs were isolated using flow cytometry. (**B**) Isolated Type II cells were subjected to epigenomic, transcriptomic, and proteomic analysis. Created via Biorender. Fernandez. J. (2025) https://BioRender.com.

The goal of the present study was to characterize genome-wide changes in cytosine methylation and hydroxymethylation, gene expression patterns, and protein abundances in the alveolar Type II epithelial cells (AECII) of A/J mice immediately after chronic exposure to LPS and after a four-week recovery period using a comprehensive multi-omics approach (Figure 1). These results contribute to the overall understanding of the epigenetic mechanisms in lung cancer and may help identify novel therapeutic targets for inflammatory lung diseases.

## 2. Results

### 2.1. Animal Studies

Our study aimed to characterize epigenetic changes in murine pulmonary Type II cells following inhalation exposure to the inflammatory agent LPS (Figure 1A). Male and female A/J mice were treated intranasally with an increasing amount of LPS once a week for three weeks and either sacrificed immediately or allowed to recover for 4 weeks. There were no fatalities associated with the LPS treatment, and no difference in weight was observed between the LPS-exposed mice and control mice. However, post-exposure observations such as “lethargy” and “hunched” were noted multiple times in most of the LPS-exposed mice throughout the exposure period.

#### 2.1.1. Histopathological Examination of Lung Tissues

Whole lungs harvested from treated and control male and female A/J mice were subjected to histopathological analysis. Pulmonary tissues of mice treated with LPS demonstrated a substantial inflammatory response characterized by chronic active pneumonitis with multifocal, locally extensive areas of inflammatory cell infiltration primarily involving the alveoli and alveolar septal walls, with a mixed inflammatory cell population comprising neutrophils and mononuclear cells consistent with lymphocytes and macrophages (Supplementary Figure S1). Septa in affected areas were variably thickened by inflammatory cells, and there was a slight degree of fibroplasia. Overall, the histopathology results were consistent with an inflammatory response to LPS in the lungs.

#### 2.1.2. Global Changes in DNA Methylation and Hydroxymethylation in Type II Alveolar Cells

**Figure 2:**
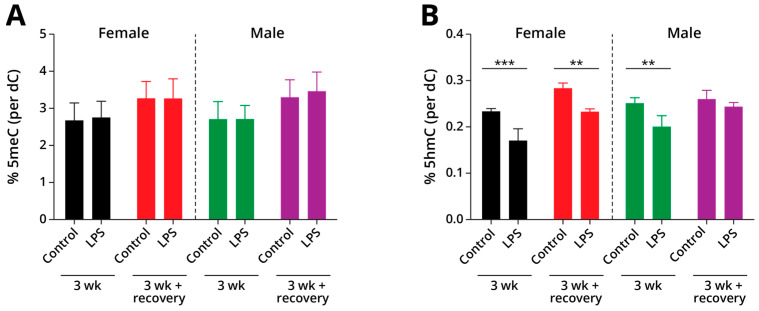
Global levels of mC (**A**) and hmC (**B**) in Type II pneumocytes of A/J mice exposed to LPS. Tissues were collected either immediately after 3-week exposure or following recovery for 4 weeks. Data are expressed as a percentage of dC and represent mean values ± SD of at least three animals. The specific treatment and duration are shown on the *x*-axis. *** = *p* ≤ 0.001; ** = *p* ≤ 0.01.

Type II alveolar cells were isolated from lung tissues of A/J mice exposed to LPS and the corresponding controls as described above. A stable isotope dilution mass spectrometry approach was used to quantify global cytosine methylation and hydroxymethylation in genomic DNA. Global genomic levels of mC were unchanged in LPS exposure groups as compared to controls (Figure 2A, female 3-week control: 3.0 ± 0.1%, female 3-week LPS: 3.1 ± 0.09%, female post-exposure control: 3.6 ± 0.1%, female post-exposure LPS: 3.6 ± 0.18%, male 3-week control: 3.1 ± 0.13%, male 3-week LPS: 3.1 ± 0.02%, male post-exposure control: 3.7 ± 0.12%, male post-exposure LPS: 3.8 ± 0.17% dC). However, global cytosine hydroxymethylation levels in genomic DNA of mice exposed to LPS exposure groups were significantly decreased in comparison with controls (female 3-week control: 0.23 ± 0.01, female 3-week LPS: 0.17 ± 0.02% dC, *p* = 0.0078; male 3-week control: 0.25 ± 0.01, male 3-week LPS: 0.2 ± 0.02% dC, *p* = 0.0134) (Figure 2B). When the animals were allowed to recover for 4 weeks post-exposure to LPS, genomic hmC values returned to baseline values in male mice but remained significantly decreased in the female mice (female post-exposure control: 0.28 ± 0.01, female post-exposure LPS: 0.23 ± 0.01% dC, *p* = 0.0133; male post-exposure control: 0.26 ± 0.02, male post-exposure LPS: 0.24 ± 0.01% dC) (Figure 2B), revealing gender-specific responses to LPS treatment.

#### 2.1.3. DNA Methylation and Hydroxymethylation Patterns

To examine the effects of inflammation on the genome-wide distribution of DNA epigenetic marks, we utilized RRBS and oxo-RRBS technologies. This approach makes it possible to detect site-specific changes in cytosine methylation and hydroxymethylation within CpG sites. These epigenetic modifications are of interest due to their well-documented role in controlling gene expression levels [23,24].

**Figure 3:**
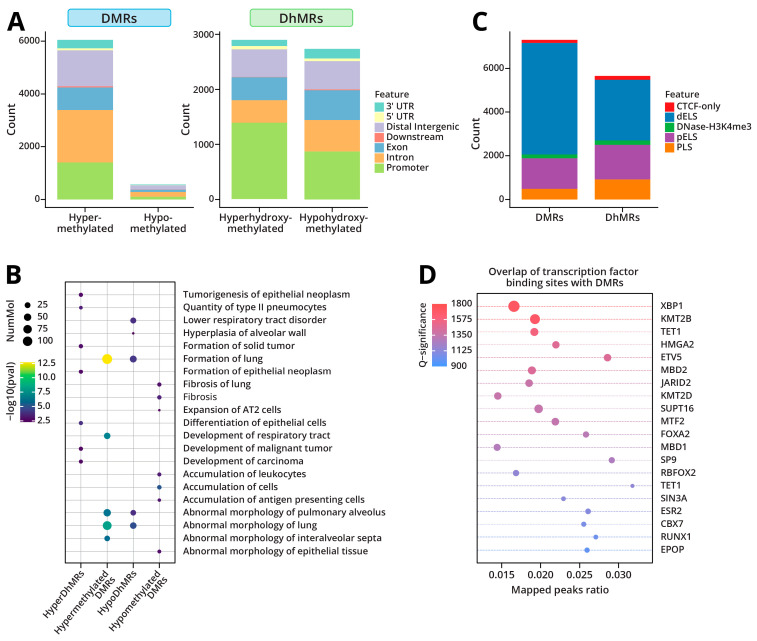
Genome-wide changes in DNA methylation and hydroxymethylation in Type II cells in response to inhalation exposure to LPS: (**A**) Genomic regions showing differential methylation (DMRs) and differential hydroxymethylation (DhMRs). (**B**) Disease and function annotations for LPS-induced DMRs and DhMRs using Ingenuity Pathway Analysis. (**C**) Overlap of DMRs and DhMRs with candidate cis-regulatory elements from the mouse ENCODE project. dELS: Distal enhancer-like; pELS: Proximal enhancer-like; and PLS: Promoter-like. (**D**) Overlap of DMRs with transcription factor binding data from mouse datasets using ReMapEnrich.

RRBS/oxoRRBS analyses of genomic DNA isolated from Type II cells of LPS-treated mice revealed 6616 CpG sites with differential cytosine methylation (DMRs) and 5655 CpG sites showing altered cytosine hydroxymethylation (DhMRs). Among CpG sites exhibiting >10% change in cytosine methylation, the majority (5174 CpGs, 91% of CpGs) showed an increase, while only 9% and 490 CpGs showed decreased methylation (Figure 3A). We found that 2684 CpGs showed decreased hydroxymethylation (47%), and 2971 CpGs had increased hydroxymethylation (53%) (Figure 3A). While the majority of methylation and hydroxymethylation changes observed following exposure to LPS returned to base levels 4 weeks post-exposure, some were persistent (Supplementary Figure S2). A pathway analysis of genes with significantly altered cytosine methylation and hydroxymethylation yielded terms associated with abnormal lung morphology, lung development and formation, and the development of lung tumors and fibrosis (Figure 3B).

Since many of the LPS-induced DMRs and DhMRs were found in intronic and intergenic regions, we employed candidate cis-regulatory element annotations from the mouse ENCODE project to determine whether these changes were occurring at cis-regulatory elements. We found that the majority of LPS-induced changes in cytosine methylation and hydroxymethylation occurred in enhancer-like regions (dELS and pELS) (Figure 3C).

DNA methylation can alter gene expression levels through the inhibition of transcription factor binding [25]. To determine whether DNA methylation changes observed upon LPS treatment could impact transcription factor binding, we overlapped the DMRs with ChIP-seq data from mouse datasets for 648 transcription factors available through ReMAP [26]. We found that the differential methylation regions overlapped with the binding sites of several key transcription factors involved in lung cancer initiation and progression such as *Etv5* and *Xbp1*, as well as factors regulating transcriptional repression and chromatin remodeling, including *Kmt2b*, *Kmt2d*, *Mbd2*, and *Tet1* (Figure 3D). Pathway analysis further predicted that transcription factor *Etv5* is a significant upstream regulator of the hypermethylated DMRs (Supplementary Table S1). Collectively, these findings suggest that LPS treatment leads to the dysregulated methylation of gene enhancer regions, which in turn can impact transcription factor binding and the corresponding changes in gene expression.

#### 2.1.4. LPS-Induced Gene Expression Changes

**Figure 4:**
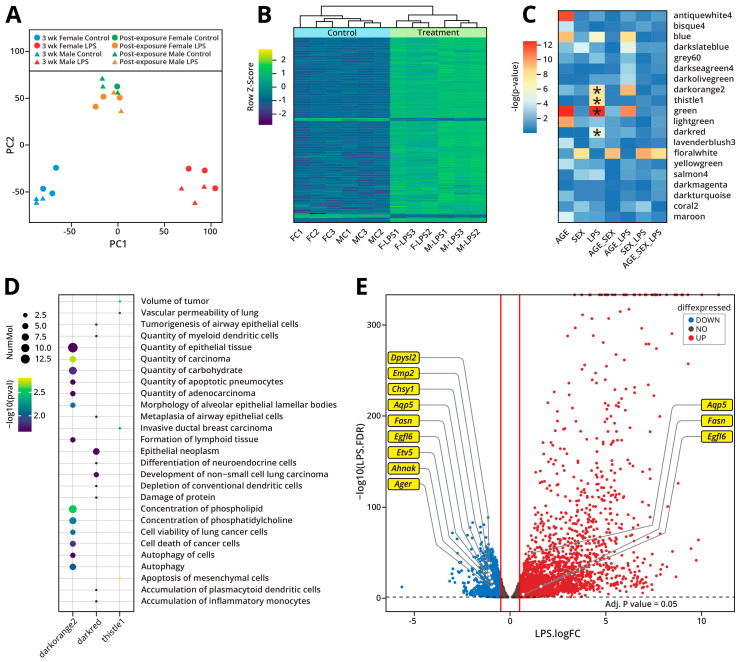
Gene expression changes in AECIIs of male and female A/J mice treated intranasally with LPS: (**A**) Principal component analysis of the gene expression changes observed in Type II cells following exposure of A/J mice to LPS for 3 weeks or 3 weeks of exposure + 4 week recovery (post-exposure). (**B**) Gene expression heat map represented as row z-score and unsupervised hierarchical clustering. (**C**) ANOVA of WGCNA module eigengene values with experimental treatment identified three modules significantly associated with LPS treatment. Starred boxes are significantly associated with LPS treatment. (**D**) Diseases and functions corresponding to the three gene modules significantly associated with LPS treatment. (**E**) Volcano plot showing differential gene expression induced by LPS exposure for 3 weeks. *Etv5* and its known targets (predicted from Ingenuity Pathway Analysis) are highlighted on the plot.

RNA-Seq analyses were conducted to identify changes in gene expression in Type II alveolar epithelial cells (AECII) of A/J mice subjected to inhalation exposure to LPS. Gene expression profiles were processed via unsupervised principal component analysis, which indicated that male and female control (Blue) and LPS-treated groups (Red) separated into distinct clusters (Figure 4A). These analyses revealed significant changes in gene expression patterns in the AECIIs of A/J mice following exposure to LPS. A total of 1797 genes exhibited significantly altered levels of expression (fold change > 2) in Type II cells of LPS-treated mice as compared to controls. Gene expression profiles observed in various samples were visualized and compared using heat maps and unsupervised hierarchical clustering (Figure 4B). As expected, the most upregulated genes in the LPS-treated mice were those coding for cytokines and chemokines such as *Il1m*, *Il1b*, *Iigp1*, and *Cxcl2* (Supplementary Figure S3A). This upregulation of proinflammatory genes is consistent with an inflammatory response of murine AECIIs to LPS. RNA-seq results for two genes, *Serpine1* and *Srgn,* were verified by qRT-PCR. The magnitude of changes in gene expression measured by RNA-Seq and qRT-PCR methods matched well for each of the genes tested, confirming our RNA-seq results (Supplementary Figure S3B).

In order to identify the patterns of gene expression changes that showed strong correlation with LPS treatment, we conducted a weighted gene co-expression network analysis (WGCNA) [27]. ANOVA of the module eigengene values identified three gene modules that were significantly associated with LPS treatment (Figure 4C). Pathway analysis of these modules (darkorange2, darkred, thistle1) identified a number of diseases and functions related to the development of lung cancer including the tumorigenesis of airway epithelial cells, the quantity of adenocarcinoma, and the development of non-small cell lung carcinoma (Figure 4D). Similar to the pathway analysis results for DMRs described above, we identified transcription factor *Etv5* as a significant upstream regulator of the darkorange2 gene module (Supplementary Table S1). This provides additional evidence for a likely role for *Etv5* in the epigenetic dysregulation following LPS exposure. Furthermore, in our differential gene expression analysis, we observed the downregulation of *Etv5* and many of its known downstream targets predicted by Ingenuity Pathway Analysis (Figure 4E).

To examine the potential reversibility of LPS-induced gene expression changes, RNA-seq analyses were repeated for cells isolated from the lungs of A/J mice from the post-exposure group. As described above, animals in this group were exposed to LPS for three weeks exactly as before but were then allowed to recover for four weeks prior to sacrifice and tissue collection. PCA revealed that, following recovery, control and post-exposure groups clustered together (Control: Green; LPS: Orange), suggesting that the gene expression differences between these two groups were very small (Figure 4A). Only 26 genes maintained significantly altered levels of expression (fold change > 2) in the post-exposure group. This indicates that, by four weeks post-exposure, the expression levels of over 98% of genes had returned to normal, indicating that the majority of LPS-induced gene expression changes are reversible.

#### 2.1.5. LPS-Induced Global Changes in Protein Abundance

**Figure 5:**
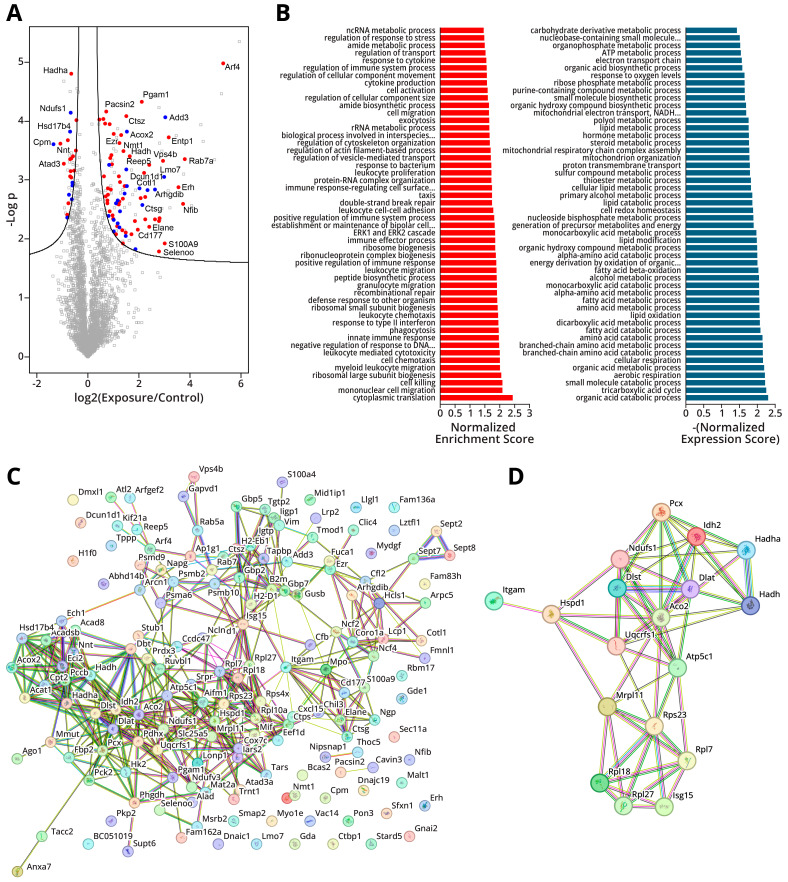
Mass spectrometry based proteomics results for gene products quantified in Type II pneumocytes of A/J mice exposed to LPS for three weeks: (**A**) Differential analysis of global mass spectrometry data annotated with lung cancer oncogenes (red) and tumor suppressors (blue). (**B**) Gene set enrichment analysis (GSEA) results for enriched genes (left, red) and depleted genes (right, blue). (**C**) Protein–protein interaction map of proteins with significantly increased or decreased abundance generated with STRING database. (**D**) Protein–protein interaction map of hub genes generated with STRING database.

TMT-based quantitative proteomics was employed to quantify global changes in protein abundance in murine Type II cells in response to intranasal exposure to LPS. NanoLC-NSI-MS/MS analysis detected 3352 protein groups across all samples. Data analysis revealed that, upon exposure to LPS, 114 proteins significantly increased in abundance, and 43 proteins significantly decreased in abundance, as shown in the volcano plot in Figure 5A. Gene set enrichment analysis (GSEA) of the altered proteins showed an enrichment in pathways consistent with an inflammatory phenotype such as the “regulation of immune system processes”, “leukocyte chemotaxis”, and “response to bacteria” alongside the depletion of pathways associated with metabolism such as “electron transport chain” and “fatty acid metabolic processes” (Figure 5B). Several enrichment pathways were consistent with an oncogenic phenotype, such as the “ERK1 and ERK2 cascade” and the “negative regulation of response to DNA damage”.

To further examine the possibility of early functional changes in the proteome following LPS exposure, proteins that showed significant alterations in their abundance were examined for any potential roles in lung adenocarcinoma. The results of this analysis are captured in Figure 5A, where proteins that have been demonstrated to have oncogenic properties in the lung are highlighted in red, and proteins with tumor suppressive capabilities are highlighted in blue. We noted that 59 proteins coded by lung cancer oncogenes showed a significant increase in abundance, while 8 tumor suppressor proteins were decreased in abundance; this is in contrast to some 22 tumor suppressors upregulated and 16 oncogenes downregulated in response to LPS treatment. This suggests a possible mechanism by which LPS-induced inflammation contributes to lung cancer development.

While many proteins have shown significant increases and decreases in abundance following LPS treatment, it is possible that there is some degree of cooperativity between them, with a few proteins controlling the expression of other gene products. To examine this possibility, we constructed a protein–protein interaction map of all the proteins that experienced a significant change in abundance using the STRING database (Figure 5C). These analyses revealed a considerable degree of interconnectivity between the affected proteins, with several serving as hubs within an elaborate network. We then ranked the node proteins by their degree of interconnectivity, with the proteins showing the highest numbers of connections—Aco2, Idh2, Pcx, Atp5f1c, Ndufs1, Hadh, Isg15, Itgam, Rps23, Dlat, Rpl27, Hadha. Mrpl11, Rpl7, Uqcrfs1, Dlst, Hspd1, and Rpl18—selected as potential hub genes (Figure 5D). Of these, Aco2 [28], Idh2 [29], Ndufs1 [30], Hadh [31], Dlat [32], Itgam [33], Dlat [34], Hadha [35], Mrpl11 [36], Dlst [34], and Hspd1 [37] show potential as oncoproteins and tumor suppressors and warrant further study for their possible role in inflammation-driven lung cancer.

#### 2.1.6. Integration of Proteomic and Transcriptomic Data

**Figure 6:**
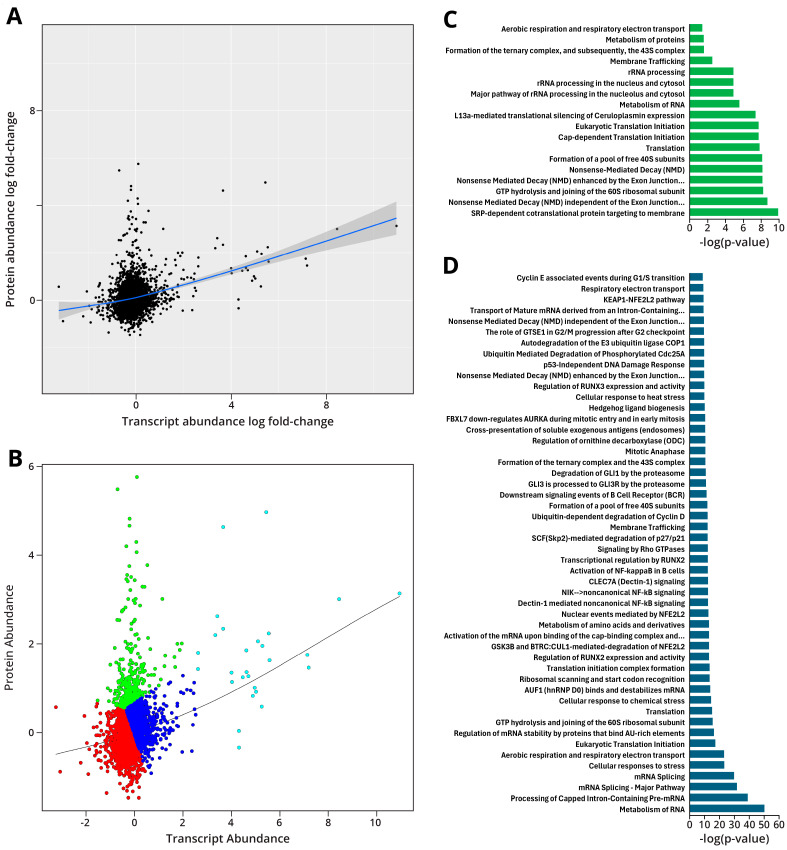
Multi-omics integration of proteomics and transcriptomics data shows limited correlation between the mRNA and protein levels: (**A**) Scatter plot of log2-fold changes in protein abundance (*y*-axis) and gene expression (*x*-axis). Pearson correlation = 0.282. Blue line indicates the best-fit correlation between the data. (**B**) K-means clustering of the scatter plot shown in Figure 6A reveals four clusters. Black line indicates a best-fit correlation between the data (**C**) Reactome pathways from the GO analysis of the green cluster in Figure 6B. (**D**) Top 50 reactome pathways from the GO analysis of the red, cyan, and blue clusters from Figure 6B.

Proteomic and transcriptomic data were compared with one another using QuanTP to determine the degree of correlation between these two datasets. Of the available proteomic and transcriptomic data, some 3222 genes were detected by both of these methods and were used for this analysis. A scatter plot of these data showed a Pearson correlation of 0.282 (Figure 6A), revealing a weak correlation between the two datasets. This is not surprising due to the independent regulatory pathways for translation, posttranslational modifications, and differing protein stability. Using k-means clustering, we found that the available data were clustered into four groups (Figure 6B), with three clusters—cyan, red, and blue—forming an approximately linear relationship between the proteomic and transcriptomic data. The fourth cluster, shown in green, consisted of genes that showed LPS-induced alteration in protein abundance with minimal change at the transcriptomic level. A GO analysis of the green cluster genes shows them to be largely involved in nonsense-mediated mRNA decay (NMD), translation, and membrane trafficking (Figure 6C). GO analysis of the linear clusters revealed the reactome pathways for stress response, NF-κB signaling, and RNA metabolism (Figure 6D), cellular activities that are expected to be affected in cells in the early stages of oncogenesis.

#### 2.1.7. Integration of the Epigenomic and Transcriptomic Data

**Figure 7:**
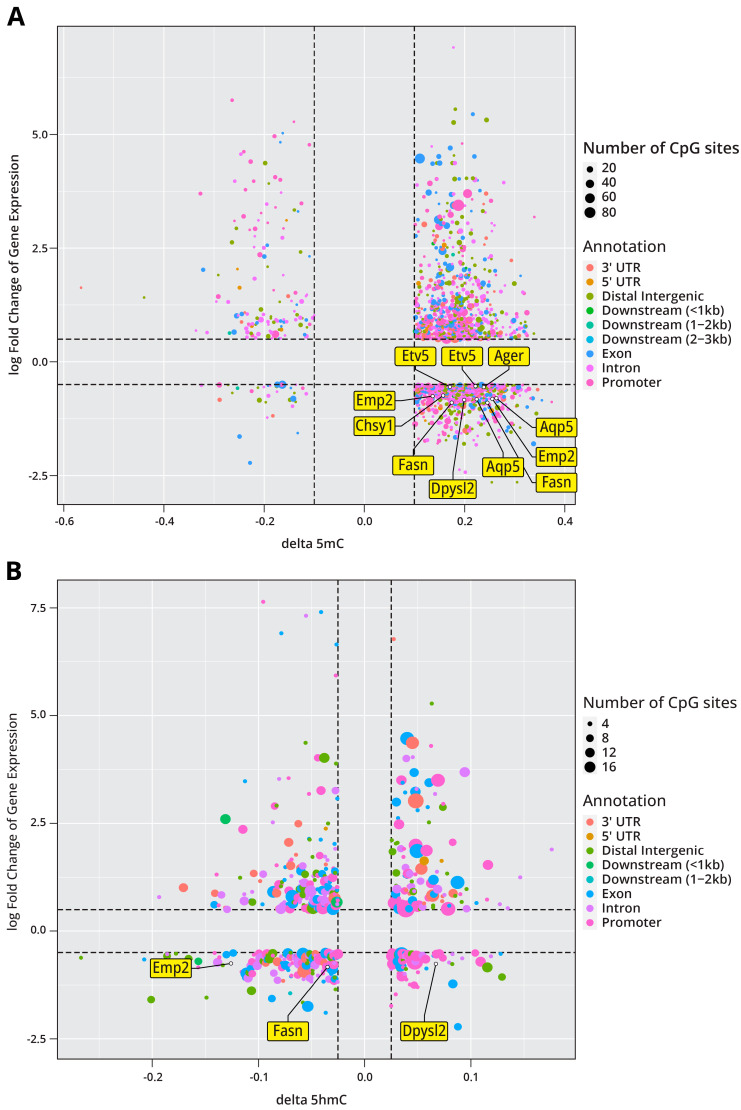
Epigenomics and transcriptomics data integration for genes affected by LPS exposure in murine Type II pneumocytes: (**A**) Log fold change in RNA transcription as a function of methylation for 490 genes found in both datasets. The transcription factor *Etv5* and its predicted downstream targets are highlighted in the plot. Black dotted lines indicate changes greater than 0.1 for delta 5 mC and fold change greater than 0.5 for transcriptomics data. (**B**) Log fold change in gene expression as a function of hydroxymethylation for 451 genes found in both datasets. Black dotted lines indicate changes greater than 0.1 for delta 5 mC and fold change greater than 0.5 for the transcriptomics data.

Because genomic cytosine methylation and hydroxymethylation modulate the levels of gene expression in mammalian cells, we additionally sought to compare the results of our epigenomic analyses with our RNA-Seq results. We found that, among the genes that showed significant changes in expression following LPS exposure, 490 also demonstrated significantly altered methylation in at least one CpG site. In plotting the log-fold change in the RNA transcription of these genes against the changes in CpG methylation, we observed that increases in DNA methylation correlated with both increases and decreases in gene expression, highlighting that DNA methylation is a complex and context-dependent epigenetic mechanism that can act as either a repressive or activation mark depending on the context (Figure 7A). Among the genes demonstrating significant expression changes, 451 also showed significant changes in 5 hmC with changes in 5 hmC also correlating with increases and decreases in gene expression (Figure 7B). Of the genes that showed a correlation between DNA methylation changes and differential gene expression, *Etv5* and several of its predicted targets (*Ager*, *Aqp5*, *Emp2*, *Fasn*, *Dpysl2*, *Chsy1*) demonstrate a significant increase in DNA methylation and decreased levels of gene expression (Figure 7A). This again highlights a role for *Etv5* in the epigenetic remodeling in response to LPS treatment.

## 3. Discussion

Smoking and chronic inflammation are known risk factors for the development of lung cancer [38,39,40,41]. Inflammatory agent lipopolysaccharide (LPS) is present in cigarette smoke and is thought to contribute to the etiology of smoking-induced lung tumorigenesis [15,42]. In an earlier study, we reported that the acute intranasal exposure of A/J mice to LPS influences gene expression in AECII, which are the target cells for lung adenocarcinoma [19]. However, the underlying epigenetic mechanisms behind inflammation-mediated changes in gene expression have remained unclear. Furthermore, previous studies have not investigated the potential reversibility of such epigenetic deregulation and did not address possible gender differences in epigenetic response to LPS-induced inflammation.

In the present work, groups of male and female A/J mice were exposed to LPS for 3 weeks, and inflammation-mediated changes in DNA methylation, hydroxymethylation, gene expression, and protein abundances were investigated in isolated alveolar Type II epithelial cells (AECII). Global levels of DNA methylation were unchanged in our exposure groups (Figure 2A). However, global cytosine hydroxymethylation levels were significantly decreased in Type II pneumocytes of male and female mice exposed to LPS for 3 weeks (Figure 2B). These results are consistent with previous studies, where LPS was shown to decrease global levels of hmC both in AECIIs and in whole lung tissues [16,19]. These changes in global hmC levels were found to be reversible in male mice, returning to baseline values after 4 weeks post-exposure; however, the levels of hmC remained significantly downregulated in Type II cells of female mice 3 weeks following exposure to LPS (Figure 2B).

RRBS/oxRRBS was employed to separately map cytosine methylation and hydroxymethylation changes in Type II cells isolated from mice exposed to LPS for 3 weeks as well as from animals allowed to recover for 4 weeks post-exposure. Our results revealed significant alterations in the DNA methylome and hydroxymethylome in mice exposed to LPS (Figure 3A,C). Interestingly, LPS-induced changes in cytosine methylation, and hydroxymethylation largely occurred in enhancer-like regions (Figure 3C). Previous work has suggested that the methylation of enhancer regions, rather than promoters, plays an important role in epigenetic regulation of tumorigenesis in lung cancer [43]. This may be due to the impact of methylation on transcription factor binding. Areas of differential methylation and hydroxymethylation after exposure to LPS were found to occur predominantly in enhancer-like regions. To investigate the potential impact of these changes on transcription factor binding, we overlapped the differentially methylated CpGs with publicly available mouse ChIP-seq data available through ReMAP [26]. We found that the regions of differential methylation significantly overlapped with binding sites of *Xbp1* and *Etv5*, transcription factors known to play a role in lung cancer tumorigenesis, suggesting that these methylation changes could interfere with transcription factor binding (Figure 3D).

The intranasal exposure to LPS for 3 weeks induced significant changes in gene expression in murine AECIIs (Figure 4A,B). As expected, exposure to LPS was associated with a significant inflammatory response, with the greatest changes in expression observed in genes coding for cytokines and chemokines (Supplementary Figure S3). Additionally, WGCNA identified three gene modules that were significantly associated with LPS treatment, when accounting for variation due to other experimental factors (Figure 4C). Pathway analysis of these gene modules was enriched in terms relating to lung cancer development and cell viability (Figure 4D). These analyses revealed a regulatory role for *Etv5* in LPS-induced epigenetic changes, as it was predicted to be a top upstream regulator of the observed gene expression changes (Supplementary Table S1). Furthermore, *Etv5* and several of its predicted downstream targets exhibited significant gene expression changes following 3-week exposure to LPS (Figure 4E). Finally, the reversibility of these gene expression changes was studied by including a post-exposure group in which the mice were exposed to LPS for 3 weeks as before and were allowed to recover for 4 weeks following the last exposure. We found that the expression levels of 98% of the affected genes had returned to baseline levels in the post-exposure group, showing that the majority of LPS-induced gene expression changes are reversible.

We next employed a mass spectrometry-based bottom-up approach to examine the global proteome changes in Type II cells of mice following intranasal LPS exposure. We found that 114 proteins were increased in abundance in the LPS-treated group, indicating a concerted cellular response following LPS-induced inflammation. GSEA analyses suggested that many of these proteins were involved in inflammation due to the enrichment of pathways such as leukocyte chemotaxis, toxin response, and innate immune response, which is consistent with our observations at the epigenomic and transcriptomic level (see above). GSEA also showed an enrichment in DNA damage repair pathways and ERK1/2 signaling, which is consistent with early oncogenesis.

Multi-omics data integration was conducted in order to examine the relationships between epigenomic, transcriptomic, and proteomic responses to LPS exposure. Integration of the proteomics and transcriptomics data showed a weak positive correlation of the two datasets (Figure 6A); the main outliers were in the cluster of genes showing large increases in protein abundance without concurrent mRNA abundance increases (Figure 6B), which were responsible for membrane trafficking and NMD responses; the increase in NMD proteins may be due to mutations at the genome level in stemming from inflammation-driven DNA damage, a process that we saw a correlation in at the mRNA and protein levels (Figure 6D). The apparent increased longevity in the mRNA of these proteins may be due to the altered control of transcription seen at the protein and mRNA levels; further experiments will be conducted to confirm these results.

Upon comparison of DNA methylation and RNA-seq data (Figure 7), we identified 490 genes where changes in DNA methylation were accompanied by altered levels of gene expression. Among these genes was *Etv5* and several of its predicted downstream targets. *Etv5* is a member of the E26 transformation-specific (ETS) family of transcription factors known to regulate the expression of genes involved in cell development, proliferation, differentiation, angiogenesis, and apoptosis. Dysregulation of these transcription factors has been shown to facilitate cell proliferation in certain cancers [44]. Furthermore, in previous work, *Etv5* was found to be essential for the maintenance of alveolar Type II cell identity [45]. Several downstream targets of *Etv5* were also found to have differential methylation or gene expression changes. *Agrn* was found to be significantly upregulated in LPS-treated animals (Figure 4E), and previous work has found *Agrn* to be overexpressed in lung adenocarcinoma and to promote lung cancer progression [46]. LPS treatment caused differential methylation of *Emp2,* leading to downregulation of its expression (Figure 7A). Dysregulated expression of *Emp2* has been observed in various cancers along with the downregulation of *Emp2,* leading to increased cancer growth and metastasis [47,48]. In addition, *Dpysl2* was found to be hypomethylated following LPS exposure, leading to its downregulation (Figure 7A). Previous work has found that *Dpysl2* has a decreased expression in lung adenocarcinoma [49]. Overall, our work suggests that LPS treatment leads to significant epigenetic changes within AECIIs. We show that these epigenetic changes largely effect the methylation and hydroxymethylation levels in gene enhancer regions, which can impact the binding of transcription factors known to play a role in lung cancer initiation and progression.

## 4. Experimental Procedures

### 4.1. Materials

Allprotect Tissue Reagent and AllPrep DNA/RNA Mini Kit were obtained from Qiagen (Hilden, Germany). Triethylammonium bicarbonate, TMT 11-plex labels, and Pierce Coomassie Bradford Protein Assay kit were purchased from Thermo Scientific (Waltham, MA, USA). SpeedBead Magnetic Carboxylate beads (45152105050250 and 65152105050250) were obtained from Millipore Sigma (Burlington, MA, USA).

### 4.2. Animal Treatments

Male and female A/J mice were purchased from Jackson Laboratories (Bar Harbor, ME, USA) and housed in specific-pathogen-free animal quarters at the AeroCore Testing Services, University of Minnesota. All animal experiments were performed according to the U.S. National Institutes of Health (NIH) Guide for the Care and Use of Laboratory Animals and were approved by the Institutional Animal Care and Use Committee, University of Minnesota (Figure 1).

### 4.3. Treatment of Mice with LPS

Male and female A/J mice (6 weeks of age) were divided into two groups (14 male/14 female mice per group). Mice in the lipopolysaccharide (LPS) treatment group were treated intranasally with LPS once a week for three weeks. LPS was dissolved in 50 µL of PBS and given as a divided dose of 25 µL in the two nostrils. In the first week of the experiment, mice were given 2.5 µg of LPS. Mice were treated with 5 µg of LPS in the second week and 7 µg of LPS in the third week. Mice in control group were treated intranasally once a week for three weeks with 50 µL of PBS and given as a divided dose of 25 µL in the two nostrils. Mice were euthanized in a CO_2_ chamber the day after their final LPS or control treatment or allowed to recover for four weeks. Lungs were harvested for alveolar epithelial Type II cell isolation as described below. The brain, heart, and liver tissues were harvested and stored at −80 °C prior to analysis.

### 4.4. Alveolar Type II Epithelial Cell Isolation

Alveolar Type II epithelial cells (AECII) were isolated according to published procedures [50]. In brief, lungs were exposed and perfused with 10 mL of cold phosphate-buffered saline (PBS). The lungs were then enzymatically digested by installing 2 mL of dispase into the lung. The lungs were then removed and incubated in an additional 2 mL of dispase for one hour. The lungs were then completely disintegrated, and the resulting cell suspension was labeled with antibodies specific for CD11c, CD11b, F4/80, CD19, CD45, and CD16/CD32. Samples from 9 mice were pooled to include three sets of lungs per sample for a total of three samples. AECIIs were isolated by negative selection and are thus identified as the unlabeled cell population [50]. AECIIs were also gated as sideward scatter high (SSC^high^) cell population, which minimizes contamination with lymphoid cells by selecting cells with a higher granularity [50]. The cells were separated by fluorescence activated cell sorting (FACS) by the University Flow Cytometry Resource at the University of Minnesota using a BD FACSAria II P07800142 (BSL2) (BD Biosciences, San Jose, CA, USA).

### 4.5. Extraction of DNA and RNA from Alveolar Type II Epithelial Cells

Following isolation via FACS, AECIIs were pelleted by centrifugation. The samples were first centrifuged for 12 min at 200× *g* and 4 °C, and the supernatant was removed, except for the bottom 1 mL, which was transferred to a 1.7 mL Eppendorf tube. Following addition of 500 µL of PBS, the tube was centrifuged for 12 min at 200× *g* at 4 °C. The supernatant was removed, except for the bottom 100 μL. Again, 1 mL of PBS was added to the tube, and the sample was centrifuged for 12 min at 800× *g* and 4 °C. After this final centrifugation, all supernatant was removed, and the cell pellet was saved for downstream analyses. A portion of the cell pellet (1.25 × 10^5^–5 × 10^5^ cells) was set aside to isolate proteins. The remaining sample was used to isolate DNA and RNA using the Qiagen AllPrep DNA/RNA Mini Kit (Qiagen, Hilden, Germany) according to the manufacturer’s instructions. DNA and RNA were then quantified using Qubit fluorometric assay (Thermo Fisher Scientific, Fairlawn, NJ, USA).

### 4.6. Extraction of Proteins from Alveolar Type II Epithelial Cells

To generate protein extract, Type II cells (1.25 × 10^5^–5 × 10^5^ cells) were transferred to 0.45 µm spin filters (Corning) and washed three times with cold PBS. Cells were lysed via the application of 50 µL lysis buffer (100 mM of TEAB pH = 8, 7 M of urea, 2 M of thiourea, 10% acetonitrile, and complete protease inhibitor tablets without EDTA) with vigorous pipetting, followed by centrifugation at 15,000 rpm for 15 min. The flow-through was collected, and protein concentrations were determined via absorbance at 280 nm using a nanodrop (Thermo Scientific, Waltham, MA, USA). Proteins were stored at −80 °C until further analysis.

### 4.7. Histopathology Examination

A subset of mice from each exposure group (*N* = 4) was used for histological analysis. The lungs were perfused, fixed in 10% buffered formalin, and embedded in paraffin. Sections were cut from paraffin-embedded tissues and stained with hematoxylin and eosin (H&E). Slides were analyzed for cellular inflammation under light microscope.

### 4.8. Reduced Representation Bisulfite Sequencing (RRBS) and Oxidative Reduced Representation Bisulfite Sequencing (Oxo-RRBS)

Genomic DNA isolated from Type II alveolar epithelial cells of control and LPS-treated A/J mice was prepared for RRBS and oxo-RRBS using the Ovation RRBS Methyl-Seq System with TrueMethyl oxBS module (NuGEN, Redwood City, CA, USA) according to the manufacturer’s protocol. Library amplification was optimized as directed using qPCR, and the libraries were amplified accordingly, followed by Agencourt bead (Beckman Coulter, Indianapolis, IN, USA) clean-up. Libraries were quantified using the PicoGreen dsDNA assay (Thermo Fisher, Waltham, MA, USA). Library size distribution was evaluated using the Bioanalyzer High Sensitivity assay (Agilent, Santa Clara, CA, USA). Paired-end sequencing (2 × 75 bp) was performed at the UMN Genomics Center using an Illumina NextSeq 550 instrument (Illumina, San Diego, CA, USA) and the 150-cycle High-Output flow cell kit (Illumina, San Diego, CA, USA).

### 4.9. RRBS and Oxo-RRBS Read Handling

RRBS and oxo-RRBS reads were screened for low-quality bases and adapter contamination with FastQC version 0.11.7 (available online: https://www.bioinformatics.babraham.ac.uk/projects/fastqc/ accessed on 10 October 2021). Sequencing reads were then trimmed of low-quality bases and adapter contaminants with TrimGalore! version 0.5.0_dev (available online: https://www.bioinformatics.babraham.ac.uk/projects/trim_galore/ accessed on 10 October 2021), with custom adapter sequences for the NuGEN Ovation RRBS MethylSeq kit. Cleaned reads were aligned to the GRCm38.p6 reference genome with Bismark version 0.19.0 [51]. Custom parameters were used to increase the sensitivity during read mapping. The resulting BAM files were then sorted, filtered of alignments with mapping quality lower than 20, and reads mapping to “blacklisted” regions of the genome (ENCODE file ENCFF547MET).

### 4.10. Methylation and Hydroxymethylation Analysis

Filtered BAM files from the RRBS and oxo-RRBS data were used to estimate methylation and hydroxymethylation levels with programs in the MethPipe version 3.4.3 suite of software [52]. BAM files were processed to generate estimated methylation levels at symmetric CpG sites in the mouse genome. Only sites covered by at least 10 sequence reads were retained for analysis. The hydroxymethylation levels were estimated using the “mlml” program in the MethPipe suite [53]. Methylation and hydroxymethylation level estimates were used to identify significantly differentially methylated regions (DMRs) and differentially hydroxymethylated regions (DhMRs). Individual CpG sites were tested for significance with the “radmeth” program in the MethPipe suite, and *p*-values were locally smoothed in 200 base pair windows with the “adjust” routine in the “radmeth” program. DMRs and DhMRs were retained as significant at the 0.05 false discovery rate and if they contained at least three significant CpG sites. DMRs and DhMRs that overlap gene bodies and promoter regions were identified with BEDTools version 2.29.0 [54]. Gene bodies and promoters that overlapped DMRs and DhMRs were used as input for a core analysis in Qiagen Ingenuity Pathway Analysis (IPA) to identify significantly enriched pathways. DMR relation within genomic regions was annotated using BEDTools v2.27.1 to the candidate cis-regulatory elements (cCREs) provided by the mouse ENCODE project [55]. To evaluate if the DMRs were located in transcription factor binding sites, ReMAP was used to assess the enrichment of transcription factors binding at these differentially methylated regions [26]. This tool overlapped the differentially methylated regions with a catalog of transcription factor binding profiles collected from public datasets of ChIP-seq experiments.

### 4.11. DNA Digestion and Enrichment of mC and hmC

Genomic DNA (0.5–2 µg) was spiked with ^13^C_10_^15^N_2_-5-methyl-2′-deoxycytidine and 5-hydroxymethyl-d_2_-2′-deoxycytidine-6-d_1_ (internal standards for mass spectrometry). The DNA was then enzymatically digested to 2’-deoxynucleosides using PDE I (55 mU), PDE II (63 mU), DNase I (28 U), and alkaline phosphatase (48 U) in a buffer containing 10 mM of Tris-HCl pH 7.0 and 15 mM of magnesium chloride and then filtered through Nanosep 10K Omega filters (Pall Corporation, Port Washington, NY, USA).

DNA hydrolysates were dried under reduced pressure and subjected to offline HPLC purification. Agilent 1100 series HPLC system was equipped with a UV detector and an automated fraction collector (Agilent Technologies, Santa Clara, CA, USA). An Atlantis T3 column (Waters, 4.6 × 150 mm, 3 µm) was eluted at a flow rate of 0.9 mL/min with a gradient of 5 mM ammonium formate buffer, pH 4.0 (A) and methanol (B). Solvent composition was changed linearly from 3% to 30% B over 15 min, increased to 80% over the next 3 min, then increased again to 96% over 1 min and maintained at 96% B for 0.5 min. Solvent composition was returned to initial conditions (3% B), and the column was equilibrated for 7 min. dC was quantified by HPLC-UV using calibration curves obtained by analyzing authentic dC standards. HPLC fractions corresponding to mC and hmC were combined, dried under reduced pressure, and analyzed by isotope dilution HPLC-ESI-MS/MS.

### 4.12. HPLC-ESI^+^-MS/MS Quantitation of Global Levels of mC and hmC

HPLC-ESI^+^-MS/MS quantitation of mC and hmC was performed using a Dionex Ultimate 3000 UHPLC (Thermo Fisher, Waltham MA, USA) interfaced with a Thermo TSQ Quantiva mass spectrometer (Thermo Fisher, Waltham, MA, USA). Chromatographic separation was achieved with a Zorbax SB-C18 column (0.5 × 150 mm, 5 µm, Agilent) eluted at a flow rate of 10 µL/min with a gradient of 2 mM ammonium formate (A) and acetonitrile (B). Solvent composition was changed linearly from 3% to 5% B over 6 min, increased to 43.5% B over the next 7 min, and maintained at 43.5% B for 1 min. Solvent composition was then returned to initial conditions (3% B), and the column was equilibrated for 7 min. Under these conditions, mC and ^13^C_10_^15^N_2_-MeC eluted at 5.8 min, while hmC and its internal standard (D_3_-hmC) eluted at 4.0 min. Quantitation was achieved by monitoring the transitions *m*/*z* 258.2 [M + H^+^] → *m*/*z* 142.1 [M-deoxyribose + H^+^] for hmC, *m*/*z* 261.2 [M + H^+^] → *m*/*z* 145.1 [M-deoxyribose + H^+^] for D_3_-hmC, *m*/*z* 242.1 [M + H^+^] → *m*/*z* 126.1 [M + H^+^] for mC, *m*/*z* 254.2 [M + H^+^] → *m*/*z* 133.1 [M + H^+^] for ^13^C_10_^15^N_2_-mC. Optimal mass spectrometry conditions were determined by infusion of authentic standards. Typical settings on the mass spectrometer were as follows: a spray voltage of 3000 V, a sheath gas of 15 units, the declustering voltage was 5 V, the RF lens was 40 V, the vaporizer temperature was 75 °C, and the ion transfer tube was maintained at 350 °C. The full width at half-maximum (FWHM) was maintained at 0.7 for both Q1 and Q3. MS/MS fragmentation was induced with collision gas pressure of 1.0 mTorr and a collision energy of 10.3 V.

### 4.13. RNA-Seq Analysis of Alveolar Type II Epithelial Cell RNA

Following extraction and quantification of RNA as described above, RNA integrity was confirmed using Agilent Bioanalyzer (Agilent, Santa Clara, CA, USA). RNA amounts for AECIIs pooled from 3 mice ranged between 300 ng and 4.1 µg. Total RNA samples were converted to Illumina sequencing libraries using SMARTer Stranded Total RNA-Seq Kit—Pico Mammalian Input (Takara Bio, Mountain View, CA, USA). RNA was reverse transcribed into cDNA, and Illumina adapters were added using PCR. Sequencing was performed on the Illumina HiSeq 2500 sequencing system using Illumina’s SBS chemistry.

### 4.14. RNA-Seq Read Processing

Raw reads were screened for low-quality bases and adapter contamination with FastQC version 0.11.7 (available online: https://www.bioinformatics.babraham.ac.uk/projects/fastqc/ accessed on 11 October 2021). Raw reads were then cleaned of low-quality bases and adapter contamination with Trimmomatic version 0.33 [56]. Surviving reads were mapped to the mouse genome build GRCm38.p6, downloaded from Ensembl release 98 with HISAT2 [57]. Known exons and splice junctions were used to aid in read mapping. The resulting BAM files were filtered of reads with a mapping quality score of less than 60, keeping only uniquely mapped reads. These filtered BAM files were then sorted by read name, and a count matrix was generated with featureCounts from the subread version 1.6.2 package [58]. Quantification was performed at the gene level. Read pairs were assigned to a gene only if both mates mapped uniquely to the same gene and if they mapped with the proper strand specificity. The scripts for performing the quality control, mapping, filtering, and generation of the counts matrix are available in the CHURP package, which is available online: https://github.com/msi-ris/CHURP (accessed on 11 October 2021) [59].

### 4.15. Expression Quantification and Filtering

The count matrix was analyzed with edgeR in the R statistical computing environment (R Core Team 2020) [60]. First, expression data for genes that were shorter than 200 bp were discarded. Then, an expression level filter was applied with the following procedure. The log2(CPM) value that corresponds to 10 fragments in the smallest library (*C*) was calculated. Then, the size of the smallest experimental group (*N*) was calculated. Genes that had a log2(CPM) value lower than *C* in *N* or more samples were removed, regardless of sample membership in any experimental group.

### 4.16. Differential Gene Expression Testing

Filtered and normalized count matrix was used to test a model that explains variations in gene expression as a function of treatment group, sex, age, and interactions among these three factors using the variancePartition package v3.21 [61]. Based on the results of the variancePartition analysis, we tested differential gene expression between treatment groups using models that accounted for the effects of sex and explicitly tested for sex-specific responses. Differential gene expression models were built and analyzed in edgeR, and significance tests were performed with a quasi-likelihood F test. Genes were labeled as significantly different at a false discovery rate of 0.05 [60].

### 4.17. Network Analysis

Gene expression values were also used to infer gene co-expression networks with the WGCNA package [27]. For input into WGCNA, raw counts were filtered in the same way as for differential gene expression testing. The filtered counts were normalized with the variance-stabilizing transformation implemented in DESeq2 [62]. A soft thresholding power of 11 was chosen for building the network because it was the lowest power where the scale-free topology model fit with an R^2^ value of at least 0.8. The network was built as an unsigned network, and modules with a correlation of greater than 0.3 were merged. The eigengene values of the resulting modules were tested for association with treatment group, age, sex, and interactions among these factors with a one-way ANOVA. Modules were labeled as significantly associated with treatment group if their Bonferroni-adjusted *p*-values were less than 0.05. Scripts to perform gene expression analysis, variance partitioning analysis, differential gene expression testing, and co-expression analysis are available upon request.

### 4.18. RNA-Seq Validation via qRT-PCR

Following extraction and quantification of total RNA as described above, its purity and integrity were confirmed using the Qubit 4 Fluorimeter (Invitrogen, Carlsbad, CA, USA). The first-strand complementary DNA was synthesized utilizing SuperScript IV VILO MasterMix (Invitrogen, Carlsbad, CA, USA) per manufacturer’s guidelines. Briefly, 8 ng of RNA was combined with ezDNAse digestion enzyme and diluted to 10 µL with 10× ezDNAse Buffer in RNAse-free water. Following incubation, 4 µL of MasterMix was added, and the solution was diluted to a total working volume of 20 µL. A C1000 Touch Thermo Cycler (Bio-Rad, Irvine, CA, USA) was used to incubate the samples per the manufacturer’s recommendations for cDNA synthesis. The resulting cDNA product was stored at −20 °C until use.

Quantitative reverse transcriptase-polymerase chain reaction (qRT-PCR) was carried out in 20 µL reaction volume on a StepOnePlus RT-PCR System (Applied Biosystems, Foster City, CA, USA) using SYBR Select Master Mix (Applied Biosystems, Foster City, CA, USA) and gene-specific primers at a final working concentration of 0.2 µM. For the PCR amplification, uracil DNA glycosylase (UDG) was activated at 50 °C for 2 min, followed by AmpliTaq activation at 95 °C for 2 min. In total, 40 cycles of amplification were completed, consisting of denaturing at 95 °C for 15 s and a combined annealing/extension at 60 °C for 60 s. All samples were normalized to an internal control of β-actin. Comparative Ct of technical triplicates was used to assess the fold change in relative mRNA expression. Fold change differences were expressed for each test group relative to their respective control groups.

### 4.19. Digestion of Proteins, Labeling, and Fractionation of Peptides

Due to the low protein amounts available for our analyses (1–5 µg of protein/sample), we utilized modified digestion, TMT labeling, and fractionation protocols. To digest proteins into peptides, single-pot solid-phase-enhanced sample preparation (SP3) beads were used [63]. Initially, protein samples were reduced via the addition of DTT (5 mM), followed by alkylation with iodoacetamide (8 mM). Following reduction and alkylation, sample volume was brought to 48 µL with phosphate-buffered saline, after which 2 µL of a washed mixture of hydrophobic and hydrophilic SpeedBead Magnetic Carboxylate beads was added to each sample, and the samples were mixed via pipetting. Next, ethanol was added to each sample to a final ethanol concentration of 70%, after which the samples were mixed again and allowed to settle for 18 min on the benchtop. Samples were then added to a magnetic rack, and the beads were allowed to settle for 2 min. The supernatant of each sample was removed and discarded, after which the pelleted beads in each sample were washed three times in 80% ethanol. The washed beads were all sonicated for 1 min to break up the beads, after which the bead pellets in each sample were resuspended in 25 µL of 20 mM TEAB (pH 8.5) supplemented with trypsin at a concentration of 1:25 enzyme to approximate protein abundance. The samples were then incubated overnight at 37 °C to digest the proteins immobilized on the beads. After overnight digestion, samples were supplemented with an additional 25 µL of trypsin solution and digested for an additional 2 h at 37 °C. Following the second digestion, sample beads were immobilized on a magnetic rack, and the supernatant was removed and retained. To extract the remaining peptides, beads were resuspended in 50 µL of 0.1% formic acid and immobilized on a magnetic rack, with the supernatants removed and pooled with the first round of supernatants. Peptides were then quantified with 280 nm absorbance on the Nanodrop, with 1 µg of aliquots set aside and dried down in the speed vac prior to TMT labeling. Peptides were labeled using TMT-11plex reagents while immobilized on C18 spin columns as detailed in Myers et al. [64] and Han et al. [65].

TMT-labeled samples were fractionated at high pH on reverse-phase stage tips in a protocol adapted from Dimayacyac-Esleta et al. [66] and Kim et al. [67] using stage tips packed with ReproSil-Pur C18-AQ slurry. Peptides were eluted using increasing amounts of acetonitrile, after which the seventeen fractions were concatenated into nine fractions and dried down for analysis.

### 4.20. HPLC-ESI^+^-MS/MS Analysis of TMT Labeled Peptides

HPLC fractionated labeled peptide samples were analyzed on an Orbitrap Fusion Tribrid Mass Spectrometer (Thermo-Fisher, Waltham, MA, USA) interfaced with an Ultimate 3000 UHPLC (Thermo-Fisher, Waltham, MA, USA). The UHPLC system was run in the nanoflow mode with a reverse-phase nanoLC column (15 cm × 250 μm) packed with 5 μm diameter Luna C18 resin. Samples were run using a 90 min gradient with 5–22% buffer B (0.1% formic acid in acetonitrile) over 71 min, followed by 22–33% over 5 min, 33–90% over 5 min, a 90% buffer B wash for 4 min, and, finally, a 90–4% decrease in buffer B over 2 min followed by a 3 min equilibration at 4% buffer B. Samples were run at a flow rate of 300 nL/min. Peptides were analyzed in the positive ion mode using a Top12 Full MS/dd-MS2 experiment with an expected chromatographic peak FWHM of 15 s. For the full MS scans, resolution was 70,000 with an AGC target of 1e6, a maximum injection time of 30 ms, and a scan range of 300 to 2000 *m*/*z*. Tandem mass spectrometry experiments were conducted at 17,500 resolution, AGC target of 5e4, maximum injection time of 50 ms, an isolation window of 2.0 *m*/*z*, and a normalized collision energy of 30. Data were collected in the centroid mode.

### 4.21. Global Proteomic Analyses

Raw mass spectrometry data were analyzed with MaxQuant v1.6.5 [68] using the murine FASTA database from Uniprot. The proteomics data were initially processed using the Reporter Ion MS/MS quantification mode with the TMT 11-plex labels selected. Carbamidomethylation of cysteine was included as a fixed modification, while oxidation of methionine, N-terminal acetylation, and phosphorylation of serine, threonine, and tyrosine were included as variable modifications. Following analysis, data were analyzed using the open-source data manipulation platform Perseus [69] to generate volcano plots. Gene ontology (GO) analyses were conducted using gProfiler [70]. Interaction networks of proteins were generated using the STRING [71] database. Proteomics data were compared with transcriptomic data generated from these same cells generated by using QuanTP [72] within the Galaxy MSI instance.

## 5. Conclusions

The present study employed a combination of isotope dilution mass spectrometry, bisulfite sequencing, oxidative bisulfite sequencing, RNA-seq, and quantitative proteomics to investigate epigenetic responses of murine alveolar Type II cells to LPS-induced inflammation. Based on our previous results [16], we hypothesized that the use of multi-omics technologies such as differential methylation and hydroxymethylation, differential transcriptomics, and differential protein abundance could identify the pathways that are integral to inflammation-driven oncogenesis in the lungs. Each of the individual-omics experiments demonstrated significant changes in response to LPS exposure, with genome-wide alterations in CpG methylation and hydroxymethylation observed alongside changes in RNA transcript and protein abundance. Integration of these multi-omics data demonstrated that exposure to LPS led to increased methylation at enhancer regions, impacting transcription factor binding and affecting gene expression changes in genes known to play a role in lung cancer tumorigenesis. Overall, our results are consistent with a model where chronic inflammation due to LPS exposure leads to changes in DNA marks, altered gene expression levels, and deregulated protein abundances that include several proteins associated with oncogenesis and tumor suppression. Collectively, these epigenetic shifts in Type II cells may contribute to their transition towards an oncogenic phenotype. Future studies should test this model further via single-cell approaches and human studies in patients with COPD.

## Data Availability

The sequencing data were deposited in NCBI GEO under accession GSE275769, and the proteomic data were uploaded under GSE275545.

## Supplementary Materials

The following supporting information can be downloaded at https://www.mdpi.com/article/10.3390/ijms26104692/s1. References [73,74] are cited in Supplementary Materials.

## Abbreviations

The following abbreviations are used in this manuscript:

AECII	Alveolar Type II epithelial cell
LPS	Lipopolysaccharide
oxo-RRBS	Oxidative reduced representation bisulfite sequencing
RRBS	Reduced Representation Bisulfite Sequencing
