# Supplementary material for: Multi-Omics Analysis of the Epigenetic Effects of Inflammation in Murine Type II Pneumocytes
Source: Int J Mol Sci. 2025 May 14;26(10):4692. doi: 10.3390/ijms26104692 (PMC12112469; doi:10.3390/ijms26104692)
Supplement: Supplementary file 1 [file ijms-26-04692-s001.zip › ijms-3602751-supplementary.pdf]

Supplementary Information for:

Multi-Omics Analysis of the Epigenetic Effects of Inflammation in Murine Type II Pneumocytes

Jenna Fernandez[1], Qiyuan Han[2], Andrew T. Rajczewski[3], Thomas Kono[4], Nicholas Weirath[5], Alexander S. Lee[6], Abdur Rahim[7] and Natalia Y. Tretyakova[8,*]

[1] Department of Medicinal Chemistry, University of Minnesota; Fernandez.Jenna@mayo.edu[a]
[2] Department of Biochemistry, Biophysics, and Molecular Biology, University of Minnesota; hanxx963@alumni.umn.edu[b]
[3] Department of Biochemistry Biophysics, and Molecular Biology, University of Minnesota; rajcz001@umn.edu
[4] Research Informatics Services, University of Minnesota; email: thomas.kono@iccb-cologne.org[c]
[5] Department of Medicinal Chemistry, University of Minnesota; weira008@umn.edu
[6] Department of Chemistry, University of Minnesota; alexanderlee2025@u.northwestern.edu[d]
[7] Department of Medicinal Chemistry, University of Minnesota; rahim032@umn.edu
[8] Department of Medicinal Chemistry, College of Pharmacy, and the Masonic Cancer Center, University of Minnesota; trety001@umn.edu
* Correspondence: trety001@umn.edu ; Tel.: 1-612-626-3432

Supplementary Figure S1. Representative pathology results from H&E-stained control PBS-treated mouse lung (**A**) and LPS-treated mouse lung (**B**). Inflammation of the lungs in the LPS-treated mouse is characterized as a chronic active pneumonitis with multifocal, locally extensive areas of inflammatory cell infiltration primarily involving the alveoli and alveolar septal walls with a mixed inflammatory cell population comprising neutrophils and mononuclear cells consistent with lymphocytes and macrophages. Pyknotic/necrotic/apoptotic cells are present in reasonable numbers. Septae in affected areas are variably thickened by inflammatory cells and there may be some slight degree of fibroplasia. The inflammatory response predominantly affects alveoli, and major and minor airways, in general, are minimally or not affected.

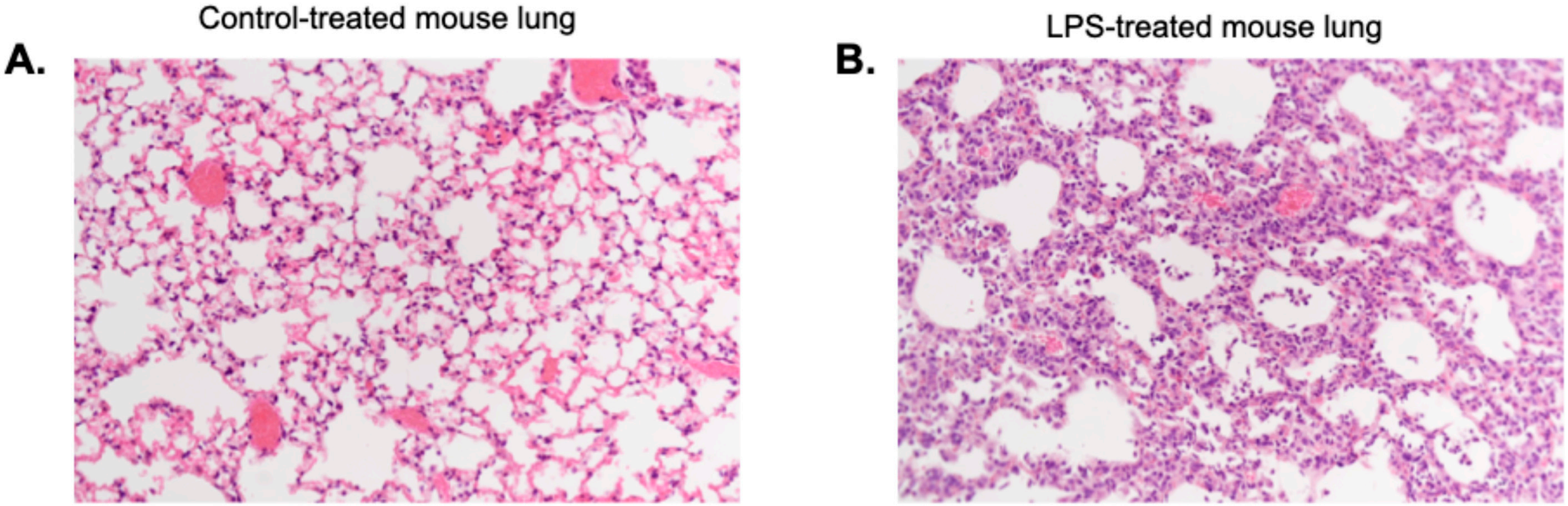

Supplementary Figure S2. Circos plot of differential methylation in the mouse genome. The outer circle represents 3 weeks of LPS exposure, while the inner circle represents 3 weeks of LPS exposure followed by four weeks of recovery. Red points indicate at least 10% increase in methylation, green points indicate at least 10% decrease in methylation, and grey points indicate intermediate levels of change. B. Circos plot of differential hydroxymethylation in the mouse genome. The outer circle represents 3 weeks of LPS exposure, while the inner circle represents 3 weeks of LPS exposure followed by four weeks of recovery. Red points indicate greater than 10% increase in hydroxymethylation, green points indicate greater than 10% decrease in hydroxymethylation, and grey points indicate intermediate levels of change.

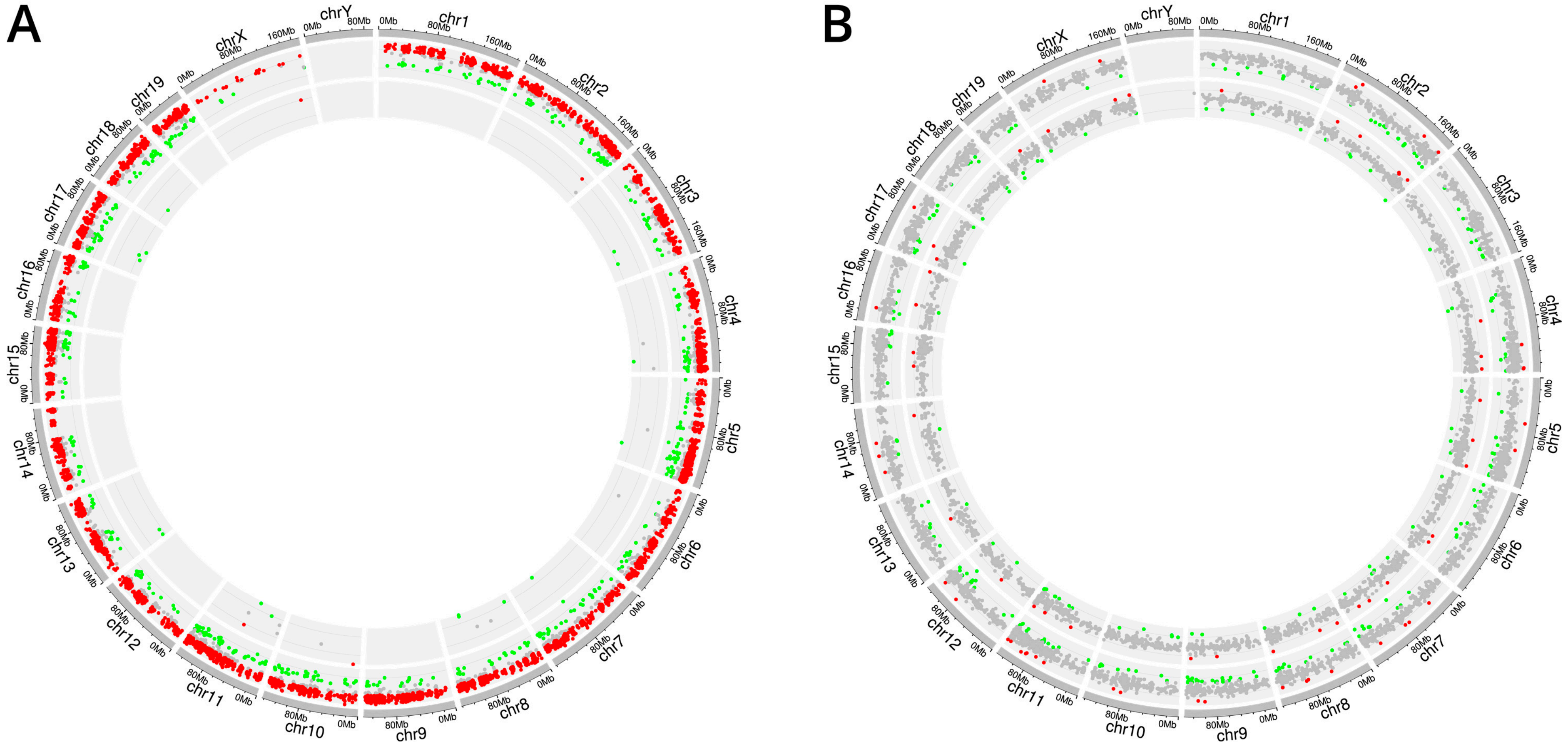

Supplementary Figure S3. Gene expression changes in Type II alveolar epithelial cells of A/J mice treated with LPS for 3-weeks. Mice were either treated for 3 weeks and then sacrificed the day after the last treatment or allowed to recover for 4-week following their last treatment (post-control and post-LPS). Fold change was calculated using the ΔΔCt method ± SD with three biological and three technical replicates. Genes (*serpine1* and *srgn*) were selected based on association with lung cancer.[1, 2] Primers used are shown in the table.

A

Gene	Fold Change	P-value
Il1m	228.65	2.90×10^{-19}
Il1b	225.27	3.88×10^{-20}
Iigp1	177.93	7.07×10^{-18}
Cxcl2	158.40	1.09×10^{-16}
Saa3	155.73	1.99×10^{-19}

B

Gene	Primer (5' → 3')	Amplicon Length
srgn	**F:** CTCGCCTTCGTCCTGGTTT<br>**R:** CCTCGATGCAGTTCGCAAAAA	112
serpine1	**F:** TTCAGCCCTTGCTTGCCTC<br>**R:** ACACTTTTACTCCGAAGTCGGT	116

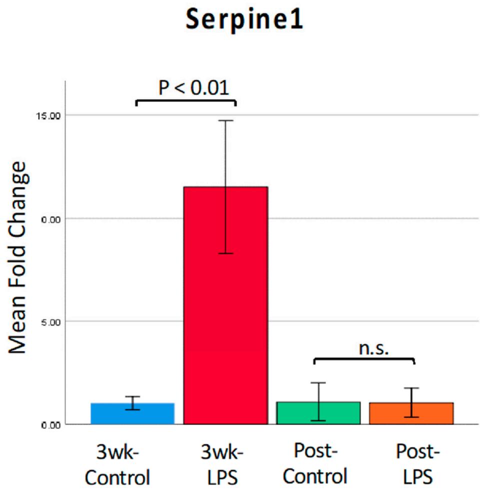

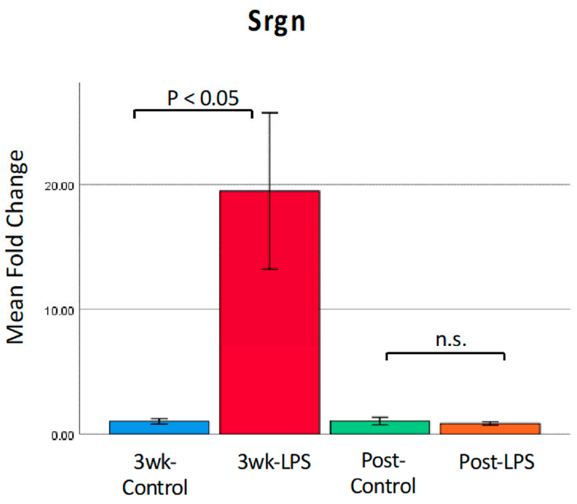

Supplementary Table S1.

Ingenuity Pathway Analysis Top Upstream Regulators	
**▾ RNA-seq darkorange2 module**	
**Name**	**p-value**
Etv5	3.25 x 10-5
Kras	1.90 x 10-3
Scap	3.26 x 10-3
Insig1	3.54 x 10-3
Wnt3a	1.32 x 10-2
**▾ RRBS hypermethylated CpGs**	
**Name**	**p-value**
Etv5	2.67 x 10-6
Ppp3r1	2.12 x 10-5
Cebpa	8.10 x 10-5
Crnde	1.38 x 10-3

References

(1) Zhu, C.; Shen, H.; Zhu, L.; Zhao, F.; Shu, Y. Plasminogen Activator Inhibitor 1 Promotes Immunosuppression in Human Non-Small Cell Lung Cancers by Enhancing TGF-Beta1 Expression in Macrophage. *Cell Physiol Biochem* **2017**, *44* (6), 2201-2211. DOI: 10.1159/000486025.

(2) Guo, J. Y.; Hsu, H. S.; Tyan, S. W.; Li, F. Y.; Shew, J. Y.; Lee, W. H.; Chen, J. Y. Serglycin in tumor microenvironment promotes non-small cell lung cancer aggressiveness in a CD44-dependent manner. *Oncogene* **2017**, *36* (17), 2457-2471. DOI: 10.1038/onc.2016.404.
